# Changes of regional cerebral blood flow after deep brain stimulation in cervical dystonia

**DOI:** 10.1186/s13550-022-00919-6

**Published:** 2022-08-09

**Authors:** Sungjin Park, Hyeonseok Jeong, Yong-An Chung, Ilhyang Kang, Seunghee Kim, In-Uk Song, Ryoong Huh

**Affiliations:** 1 Department of Neurology, Incheon St. Mary’s Hospital, College of Medicine, The Catholic University of Korea, Seoul, South Korea; 2 Department of Nuclear Medicine, Incheon St. Mary’s Hospital, College of Medicine, The Catholic University of Korea, Seoul, South Korea; 3 Department of Radiology, Incheon St. Mary’s Hospital, College of Medicine, The Catholic University of Korea, Seoul, South Korea; 4 Ewha Brain Institute, Ewha Womans University, Seoul, South Korea; 5 Department of Neurosurgery, Incheon St. Mary’s Hospital, College of Medicine, The Catholic University of Korea, Seoul, South Korea

**Keywords:** Cervical dystonia, Deep brain stimulation, Regional cerebral blood flow, Single-photon emission computed tomography

## Abstract

**Introduction:**

Cervical dystonia is considered as a network disorder affecting various brain regions in recent days. Presumably, deep brain stimulation (DBS) of the internal segment of globus pallidus (GPi) may exert therapeutic effects for cervical dystonia through modulation of the aberrant brain networks. In the present study, we investigated postoperative regional cerebral blood flow (rCBF) changes after GPi DBS using single-photon emission computed tomography (SPECT) to identify significant activity changes in several relevant brain areas of cervical dystonia patients.

**Methods:**

A total of 9 patients with idiopathic cervical dystonia were recruited, and SPECT scans were conducted at baseline and 3 months after the bilateral GPi DBS. Voxel-wise changes of rCBF were analyzed using Statistical Parametric Mapping. Symptom severity of dystonia was measured using Toronto Western Spasmodic Torticollis Rating Scale (TWSTRS) at the baseline, and 1 week, and 3 months after GPi DBS.

**Results:**

At the 3-month follow-up after DBS, rCBF was increased in the left pons and right postcentral gyrus and decreased in the left middle frontal gyrus, left cerebellum, right putamen and pallidum, and left thalamus (*p* < 0.001). Severity of cervical dystonia assessed by TWSTRS was significantly decreased at 1-week and 3-month follow-up (*p* = 0.004).

**Conclusions:**

Clinical improvement of cervical dystonia after GPi DBS may be accompanied by rCBF changes in several brain areas of the cortico-basal ganglia-cerebellar network which are important for sensorimotor integration.

## Introduction

Cervical dystonia, as the most typical form of idiopathic focal dystonia, is characterized by abnormal head posture with pain or jerky movements due to involuntary muscle contractions of neck. To date, the first-line treatment for focal dystonia is botulinum toxin injections generating clinical response with peripheral chemo-denervation of the affected muscles. Meanwhile, deep brain stimulation (DBS) of the internal segment of the globus pallidus (GPi) has shown therapeutic efficacy in various types of idiopathic dystonia including cervical dystonia [1].

However, many reports have recently addressed that dystonia may be a network disorder involving the cortico-subcortical circuits and various brain areas [2]. DBS exerts its therapeutic effects by modulating the brain networks relevant to the target and the GPi is the principal output structure of the basal ganglia as part of the sensorimotor, associative, and limbic system [3, 4] Considering this, changes in the network involving the GPi may be action mechanisms of the GPi DBS on cervical dystonia. However, the neural correlates underlying clinical improvement after the GPi DBS treatment for cervical dystonia remain unknown.

This study evaluated changes in regional cerebral blood flow (rCBF) after the bilateral GPi DBS in patients with idiopathic cervical dystonia using single-photon emission computed tomography (SPECT).

## Methods

### Participants and study design

A total of 9 patients with idiopathic cervical dystonia were recruited at Incheon St. Mary’s Hospital (Incheon, South Korea) between April 2015 and June 2016. Patients diagnosed as idiopathic cervical dystonia by experienced neurologists were eligible for this study. Exclusion criteria were family history of dystonia, structural cerebral lesions other than small vessel disease, significant cognitive dysfunction, and other neurological or psychiatric comorbidities.

SPECT scans were conducted at baseline (approximately 2 months before GPi DBS) and 3 months after the bilateral GPi DBS, and symptom severity of the dystonia was measured 3 times; baseline, 1 week, and 3 months after GPi DBS, using Toronto Western Spasmodic Torticollis Rating Scale (TWSTRS). This study was approved by the Institutional Review Board of the Incheon St. Mary's Hospital and informed consent was obtained from all participants.

### Deep brain stimulation

Mild conscious sedation and local anesthetic were used to perform the surgical procedure. The initial coordinates of the GPi were assigned based on the anterior and posterior commissures: 2 mm anterior and 4 mm inferior to the midcommissural point and 22 mm lateral to the third ventricle. Based on the above indirect targeting measures, direct targeting was implemented based on the magnetic resonance imaging performed on the day of surgery to compensate for individual variation. The entry point and final trajectory were established using the planning software to avoid the ventricles, sulci, and vessels along the electrode trajectory. The burr hole was placed about 1 cm anterior to the coronal suture and 3.5–4 cm from the midline. Microelectrode was recorded beginning 5 mm above the target using 3 concentric bipolar tungsten microelectrodes which were driven simultaneously by a Elekta Microdrive at incremental depths of 0.5 mm until 2 mm above the target and then 0.2 mm depth. The ventral and posterior borders of the GPi were located and confirmed by intraoperative electrophysiology by Lead point system. For the GPi, the trajectory was planned to be lateral to the ventricle, to traverse the posterior GPi, and to terminate just above the optic tract. The GPi neuronal activity was confirmed using microelectrode recordings and macrostimulation was used to to optimize the final target. The final electrode placement was based on intraoperative effect, which was further refined by macrostimulation. The clinical responses and side effects were confirmed using intraoperative limb and jaw movements, as well as speech and vision testing. Medtronic quadripolar or Abbott or Boston scientific directional electrodes were implanted bilaterally. At a subsequent surgery, the DBS electrodes were connected to an implantable pulse generator. The accuracy of electrode placement in the GPi and postoperative complications such as intracerebral hemorrhage were assessed using postoperative nonenhanced brain computed tomography imaging.

After 2–3 days of the surgery, the DBS power was turned on according to the patient's condition, mostly starting with a low voltage of 1.5 V, a frequency of 130 Hz, and a width of 60 pulse. After that, by observing the patient's condition, the amplitude was increased and the frequency and pulse width were also finely adjusted. Most of the amplitude was maintained at about 3.5 V, and the frequency and pulse width were maintained around ± 5 from the initial setting value. DBS was also working during the tracer uptake.

### Brain SPECT imaging

Brain SPECT data were obtained using a dual-headed gamma camera (Discovery NM630; GE Healthcare, Milwaukee, WI, USA) with a low-energy fan-beam collimator. Patients were injected with 555–740 MBq of technetium-99 m hexamethylpropylene amine oxime (^99m^Tc-HMPAO) and rested for approximately 40 min prior to scanning. Images were acquired by rotating the camera a total of 720° at 6-degree intervals at a rate of 12 s per frame, while patients were in a supine resting position. Continuous transaxial images were reconstructed as follows: matrix = 128 × 128, field of view = 250 mm, pixel size = 1.95 × 1.95 mm, slice thickness = 2.08 mm, 20% symmetric energy window at 140 keV. To reduce noise, the standard ordered subset expectation maximization algorithm (6 iterations and 10 subsets) and a Butterworth filter (cut-off frequency = 0.5, power = 10) were applied.

SPECT images were preprocessed and analyzed using Statistical Parametric Mapping 12. The images were spatially normalized to the standard SPECT template, resliced to 2.0 mm isotropic resolution, and smoothed with a 12 mm full width at half maximum Gaussian kernel. Global normalization was conducted using proportional scaling. A whole-brain voxel-wise paired t-test was performed to evaluate changes of rCBF between the baseline and follow-up. The voxel-level significance threshold was set at *p* < 0.001 with the cluster-level threshold of 20 voxels.

### Statistical analysis

Changes of TWSTRS total scores were analyzed with Wilcoxon signed‐rank tests. For each cluster with significant changes in rCBF, Spearman's rank correlations were assessed between 3-month changes of rCBF and those of TWSTRS total scores. A *p* value of < 0.05 was considered significant. Statistical tests were carried out using STATA version 16 (StataCorp., College Station, TX, USA).

## Results

A total of 9 patients with cervical dystonia underwent GPi DBS and completed baseline and follow-up assessments. The mean age was 54.7 ± 10.7 years and 8 patients were female.

**Fig. 1:**
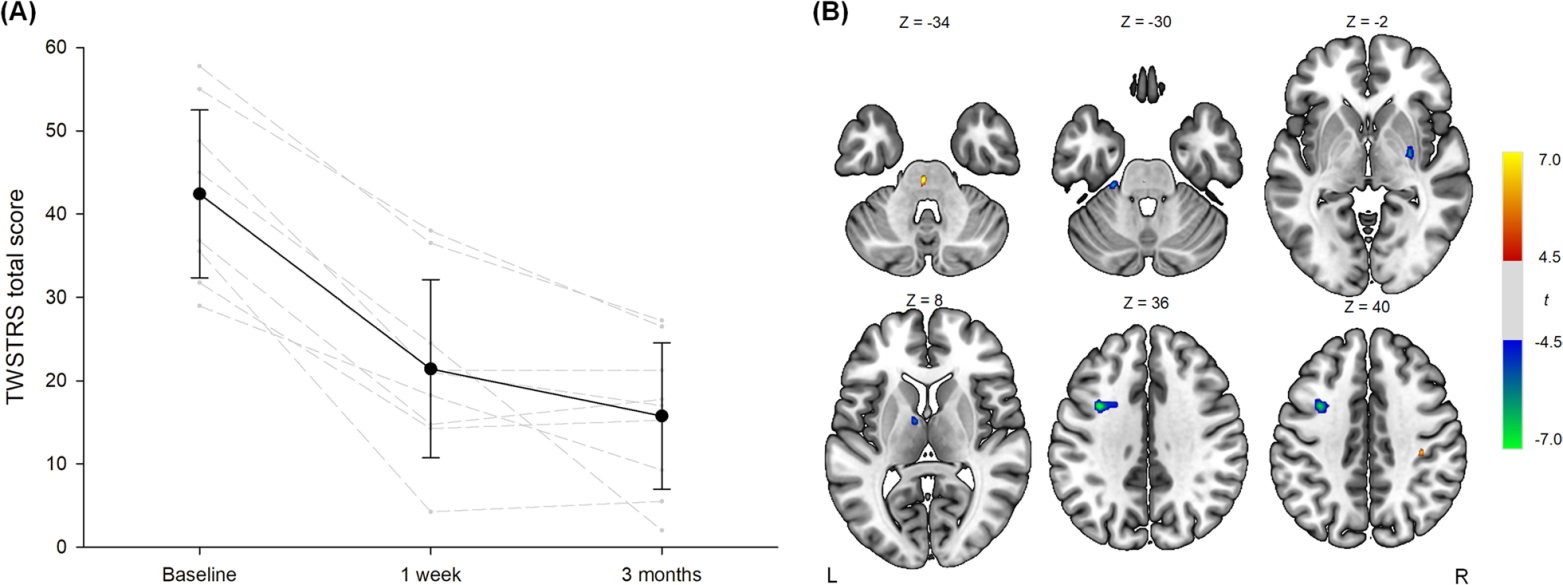
**A** Changes of Toronto Western Spasmodic Torticollis Rating Scale (TWSTRS) total scores in patients with dystonia after pallidal deep brain stimulation. The gray dashed lines indicate the individual scores, and the black solid line and error bars show the mean scores and standard deviations, respectively. **B** Increases (red-yellow) and decreases (blue-green) of regional cerebral blood flow in patients with dystonia after pallidal deep brain stimulation. The axial brain slices with significant clusters are shown in neurological convention. The color bar indicates t-values at each voxel. L, left; R, right

There were significant decreases in TWSTRS total scores at 1-week (21.4 ± 0.7, *z* =  − 2.67, *p* = 0.004) and 3-month follow-up assessments (15.8 ± 8.8, *z* =  − 2.67, *p* = 0.004) compared to baseline evaluation (42.4 ± 10.1) (Fig. 1A). However, changes of TWSTRS scores between 1-week and 3-month did not reach statistical significance (*z* =  − 1.54, *p* = 0.15).

In the SPECT analysis, rCBF was increased in the left pons and right postcentral gyrus and decreased in the left middle frontal gyrus, left cerebellum, right putamen and pallidum, and left thalamus (*p* < 0.001) (Table 1 and Fig. 1B). There were no significant correlations between 3-month changes of rCBF and those of TWSTRS total scores.

**Table 1:** Clusters with significant changes of regional cerebral blood flow in patients with dystonia after pallidal deep brain stimulation

Brain regions	Cluster size (voxels)	*t*	*z*	*p*	MNI coordinates (x, y, z)
*Increased regional cerebral blood flow*					
Left pons	24	9.75	4.41	< 0.001	− 2, − 26, − 34
Right postcentral gyrus	23	6.89	3.84	< 0.001	36, − 26, 40
*Decreased regional cerebral blood flow*					
Left middle frontal gyrus	154	7.96	4.08	< 0.001	− 34, 6, 36
Left cerebellum	22	6.93	3.85	< 0.001	− 24, − 30, − 30
Right putamen, pallidum	38	6.24	3.66	< 0.001	30, − 10, − 2
Left thalamus	20	6.10	3.63	< 0.001	− 8, − 4, 8

*MNI* Montreal Neurological Institute

## Discussion

This study showed that DBS significantly improved dystonia symptoms and changed rCBF in several brain regions including the prefrontal cortex, postcentral gyrus, basal ganglia, thalamus, cerebellum, and pons. The large-scale network derangement of the brain areas has been regarded as the pathological mechanism of dystonia. A meta-analysis of functional neuroimaging studies of dystonia has demonstrated abnormal activities in the brain regions related to sensorimotor integration and high order motor planning, such as the primary sensorimotor cortex and cerebellum [5]. In addition, functional network studies have reported higher intrinsic functional connectivity of the postcentral gyrus [6] and lower degree centrality of the lentiform nucleus in patients with cervical dystonia [7]. Our results suggest that the treatment effect of DBS may be driven by functional changes in the brain areas of the cortico-basal ganglia-cerebellar network.

The main causes of dystonia were considered as the loss of motor inhibition, driven by hyperactivation of the direct pathway from basal ganglia. However, the sensorimotor integration has recently gained attention for its clinical significance in pathophysiology of dystonia [8]. For instance, sensory symptoms often precede motor symptoms and mild sensory deficits are commonly identified in patients with various types of dystonia (9). Sensory trick, easing of hyperkinetic motor symptoms upon touching relevant areas, is also considered as a sign suggesting the defects in sensorimotor function as contributing factor of dystonia [10]. Moreover, somatosensory temporal discrimination threshold was reported to be abnormal in several types of focal dystonia, regardless of the distribution and severity of motor manifestations [11]. The cholinergic system, basal ganglia, and cerebellum were suggested as the underlying neuroanatomic substrates in previous literature [12]. This is further supported by changes of rCBF found in the current study.

On the other hand, our results also correspond with more classical view emphasizing abnormalities in activity of the basal ganglia and efferent connections from the basal ganglia to the thalamus and brainstem in dystonia. Collectively, in accordance with earlier studies, rCBF alterations in our study support that dystonia may be a multi-system network disorder involving the motor and somatosensory system. Further studies should be conducted to further elucidate the pathophysiology.

The clinical symptoms assessed by TWSTRS showed improvement at both 1-week and 3-month follow-up, mostly in the first week after the GPi DBS. Previous studies in patients with dystonia suggested that the immediate improvement may occur within 1 week after the surgery and the clinical benefits are mostly from the one to two months after GPi DBS [13, 14]. In studies with longer follow-up periods, sustained improvement of dystonia symptoms can be found over 12 months [13, 14]. Thus, changes of the clinical symptoms may be significant if additional follow-up assessments are conducted several months later.

Several limitations of this study should be addressed. First, our results are preliminary due to small sample size which may partly explain small cluster sizes found as significant in the current study. Second, healthy controls were not recruited due to ethical issues. Third, the follow-up period was relatively short. It cannot be ruled out that we observed beginning of the network alterations driven by DBS and further significant changes may be discovered afterwards. Some studies have suggested that improvement of motor symptoms may be relatively delayed for several months in contrast to rapid reductions in pain after GPi DBS [13]. Therefore, rCBF patterns at the long-term follow-up more than 3 months may demonstrate aspects that are different from our findings.

In conclusion, our findings suggest that therapeutic effect of GPi DBS may be associated with changes of activities in the multiple brain areas in the cortico-basal ganglia-cerebellar network. Further longitudinal studies with larger samples and longer follow-up periods are needed to elucidate more detailed neurobiological mechanisms of DBS in cervical dystonia. In addition, associations between rCBF and symptom improvement should be explored to find out which brain regions are more important for the DBS-related functional changes and whether SPECT imaging could be useful to predict the therapeutic effects of DBS in cervical dystonia.

## Data Availability

The datasets presented in this article are not readily available because the IRB has restrictions on sharing datasets. Requests to access the datasets should be directed to I.U.S. (siuy@catholic.ac.kr).

## Abbreviations

99mTc-HMPAO: Technetium-99 m hexamethylpropylene amine oxime
DBS: Deep brain stimulation
GPi: Globus pallidus
rCBF: Regional cerebral blood flow
SPECT: Single-photon emission computed tomography
TWSTRS: Toronto Western Spasmodic Torticollis Rating Scale
